# Lung cancer in never-smoking females: epidemiology, risk factors and screening

**DOI:** 10.3389/fpubh.2026.1834538

**Published:** 2026-05-25

**Authors:** Yumeng Ding, Huanqing Tao, Chen Zhu, Huizhang Li, Yanting Zhang, Yihui Du, Lingbin Du, Weiwei Gong, Le Wang

**Affiliations:** 1 Department of Cancer Prevention, Zhejiang Cancer Hospital, Hangzhou, China; 2 School of Public Health, Nanjing Medical University, Nanjing, China; 3 Department of Epidemiology and Health Statistics, School of Public Health, Guangdong Medical University, Dongguan, China; 4 Department of Epidemiology and Health Statistics, School of Public Health and Nursing, Hangzhou Normal University, Hangzhou, China; 5 Zhejiang Provincial Centre for Disease Control and Prevention, Hangzhou, China

**Keywords:** epidemiology, lung cancer, never-smoker, risk factors, screening

## Abstract

Although smoking is a well-established risk factor, lung cancer in never-smokers exhibits distinct epidemiological and biological characteristics. Globally, approximately two-thirds of lung cancer cases in never-smokers occur in females, with a higher prevalence observed in Asian populations. Understanding these epidemiological patterns and specific risk factors holds significant implications for informing effective prevention and control strategies. In addition, low-dose CT screening for never-smoking females remains controversial due to insufficient evidence to support its benefits and unresolved concerns about overdiagnosis. This review provides a comprehensive overview of lung cancer among never-smoking females, synthesizing current evidence on its epidemiology, risk factors, and screening practices, thereby elucidating the multifaceted nature of lung cancer in this population and highlighting its implications for prevention, risk stratification, and future research priorities.

## 1 Introduction

Lung cancer remains the leading cause of cancer incidence and mortality worldwide and shows marked sex-specific divergence. While incidence rates have steadily declined in males, they have paradoxically increased in females (1, 2). This trend is especially pronounced in Asia, where Chinese females have experienced an annual increase of 8.3% despite a smoking prevalence below 2% (3, 4). The discordance between minimal tobacco exposure and rising incidence suggests that non-smoking-related environmental and biological factors play major roles in this population. Lung cancer in never-smokers (LCINS) is generally defined as lung cancer occurring in individuals who have smoked fewer than 100 cigarettes in their lifetime. In this review, we focus on lung cancer in never-smoking females (LCINSF).

Although smoking is the dominant global risk factor for lung cancer, its population attributable fraction (PAF) varies substantially by region and sex. In industrialized countries such as the United Kingdom and the United States, smoking accounts for more than 80% of lung cancer cases in both sexes, whereas in China it accounts for 57.5% of male cases and only 12.5% of female cases (5). This low attributable fraction in Chinese females highlights the importance of alternative risk factors, including secondhand smoke (SHS), ambient fine particulate matter with an aerodynamic diameter of ≤2.5 μm (PM2.5), household air pollution from cooking fumes and coal use, pre-existing pulmonary disease, hormonal and reproductive factors, family history of lung cancer, and occupational exposures (6–37). However, the clustering, interactions, and dose–response relationships of these factors remain incompletely characterized.

Low-dose computed tomography (LDCT) reduces lung cancer mortality by 20–24% in heavy smokers (38, 39), but its value in LCINSF remains uncertain. Observational studies, mainly from Asia, suggest that LDCT screening in never-smokers yields substantial detection rates and a high proportion of early-stage disease (40–46). However, whether this translates into mortality benefit is unclear, and concerns regarding overdiagnosis and overtreatment remain substantial (47–50).

Lung cancer in never-smoking females has received increasing attention in recent years, and related evidence is expanding rapidly. Several reviews have examined lung cancer in women or in nonsmoking populations more broadly (51, 52). However, a review focused specifically on never-smoking females remains valuable for three reasons. First, this population lies at the intersection of sex-specific susceptibility and non-smoking-related exposures. Second, important risk factors in this group, such as household air pollution, cooking-related exposures, and family history, vary substantially across regions and are often not examined within an integrated female-specific framework. Third, screening in never-smoking females has become a major clinical and public health controversy because higher detection rates may coexist with uncertain mortality benefit and a substantial risk of overdiagnosis. Accordingly, this review integrates epidemiological trends, major risk factors, screening evidence, and public health implications in never-smoking females, with particular attention to sex-specific susceptibility, geographic heterogeneity, and translational relevance.

This article was conducted as a structured narrative review. Data on smoking prevalence and lung cancer incidence were obtained from the World Health Organization and GLOBOCAN 2022/Cancer Today databases, respectively (4, 53). For evidence on epidemiology, risk factors, and screening, we searched the PubMed database for relevant English-language publications from January 1, 2000 to October 15, 2025. The search strategy combined terms related to lung cancer (“lung cancer” OR “lung neoplasms”), smoking status (“never-smoker” OR “nonsmoker”), sex (“female” OR “women”), and topics of interest including epidemiology, risk factors, and screening (“screening,” “low-dose computed tomography,” “LDCT,” “secondhand smoke,” “air pollution,” “family history,” “occupational exposure,” and “cooking oil fumes”).

We prioritized original studies, meta-analyses, pooled analyses, major cohort studies, randomized trials, and authoritative reports that focused on never-smoking females or provided sex-specific or never-smoker subgroup data. Studies were excluded if they focused exclusively on active smokers, did not provide relevant subgroup information, were duplicate publications, or had limited relevance to the scope of this review. We also manually screened the reference lists of key articles to identify additional relevant studies.

As this was not a formal systematic review, we did not apply a standardized risk-of-bias assessment tool. However, in synthesizing the evidence, we gave greater weight to studies with robust designs, larger sample sizes, consistent findings, and clear exposure definitions, while also considering geographic context and potential confounding. As a structured narrative review, this article aims to integrate epidemiological patterns, major risk factors, screening controversies, and public health implications in never-smoking females.

## 2 Epidemiology

**Figure 1:**
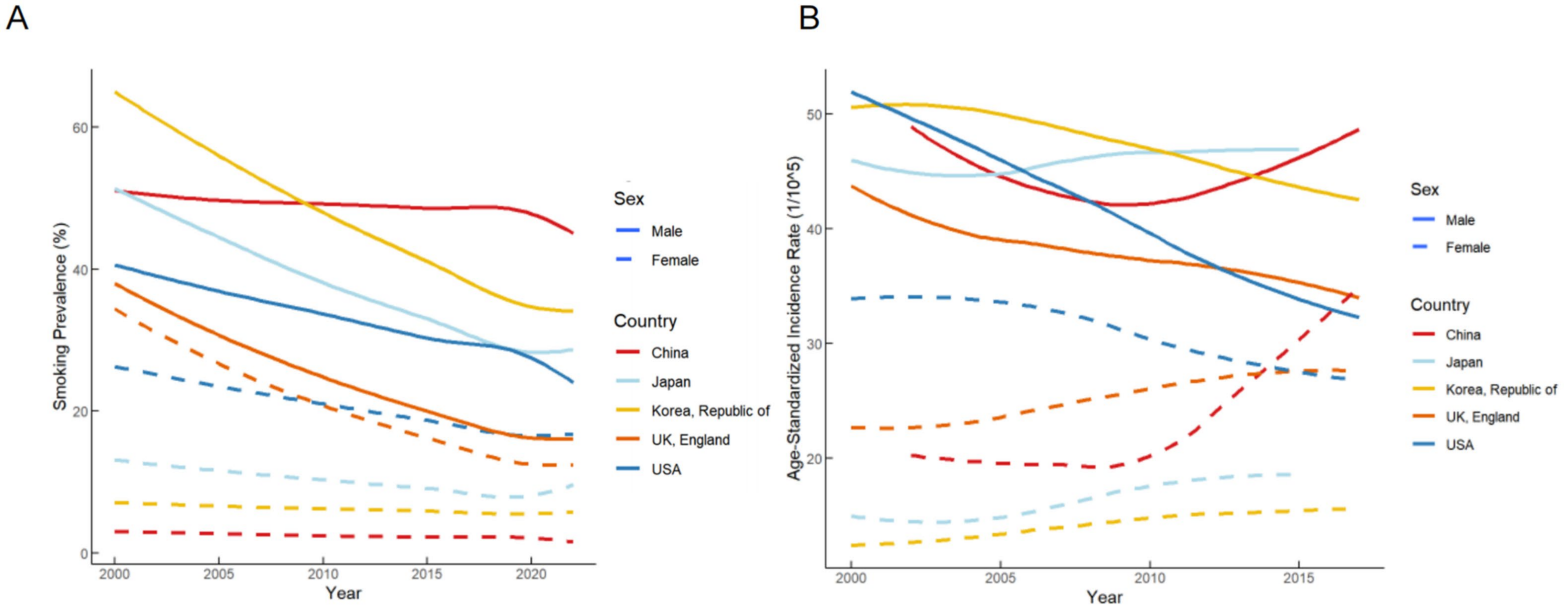
Smoking prevalence and age-standardized incidence rate. Solid lines represent males; dashed lines represent females. **(A)** Smoking prevalence in China, Japan, Republic of Korea, UK, USA from 2000 to 2022, by sex. The data are derived from World Health Organization (4). **(B)** Age-standardized incidence rate in China, Japan, Republic of Korea, UK, USA from 2000 to 2017, by sex. The data are derived from Global Cancer Observatory (53).

Globally, approximately 25% of lung cancer cases occur in never-smokers, and about two-thirds of these cases are in females, a pattern particularly evident in Asia (5). Data from the Global Cancer Observatory and WHO 2022 regional smoking prevalence (Figure 1) show that males consistently have higher smoking rates and lung cancer incidence than females (4, 53). In males, smoking prevalence generally parallels age-standardized incidence rates (ASIRs), whereas in females a “low smoking prevalence–high ASIR” pattern is evident. This is particularly striking in China, where female smoking prevalence remained below 3.0% from 2000 to 2022 (1.6% in 2022), yet the ASIR of lung cancer in females reached 30.3 per 100,000 in 2022, nearly identical to that in the United States (30.4 per 100,000), despite a much higher smoking prevalence among U.S. females (3, 4, 53). Similar discrepancies have been reported in Korea and the United Kingdom, supporting an important contribution of non-smoking-related factors. This is consistent with smoking-attributable PAFs, which are much lower in Asian females than in Western females (54).

Significant geographical disparities characterize the incidence of lung cancer among never-smoking females (NSF). Epidemiological data reveal striking differences between Asian and Western populations: while never-smokers constitute <20% of female lung cancer cases in the US and UK, this proportion surpasses 80% in China (5). Temporal trends further highlight regional variations. ASIR among NSF in Western populations remained stable over the past two decades (55, 56), whereas Asian populations exhibited a pronounced upward trajectory. Japanese registry data demonstrate a dramatic increase in the proportion of NSF among female lung cancer cases, from 11.2% (1974) to 85.8% (2002) (57). Correspondingly, a Korean hospital-based study reported that never-smokers accounted for 83.7% of female lung cancer cases by 2014 (58). Notably, China presents a paradoxical epidemiological pattern, despite stable low smoking prevalence among females, the ASIR of lung cancer surged from 20.0 to 30.3 per 100,000 during the same period (3). This observed epidemiological shift may reflect either true incidence escalation or relative increases due to declining smoking-attributable cases. Further epidemiological studies with granular smoking-status stratification are urgently needed to clarify these trends.

Adenocarcinoma represents the predominant histological subtype of lung cancer among NSF. According to the International Agency for Research on Cancer (IARC), adenocarcinoma comprises 59.7% of female lung cancer cases globally, far exceeding squamous cell carcinoma (17.1%) (59). This predominance is especially marked in Asia: 91.1% in Chinese NSF and 77% in U.S. Asian NSF (11, 60). The consistent adenocarcinoma preponderance in NSF suggests an etiology distinct from smoking-related subtypes.

**Figure 2:**
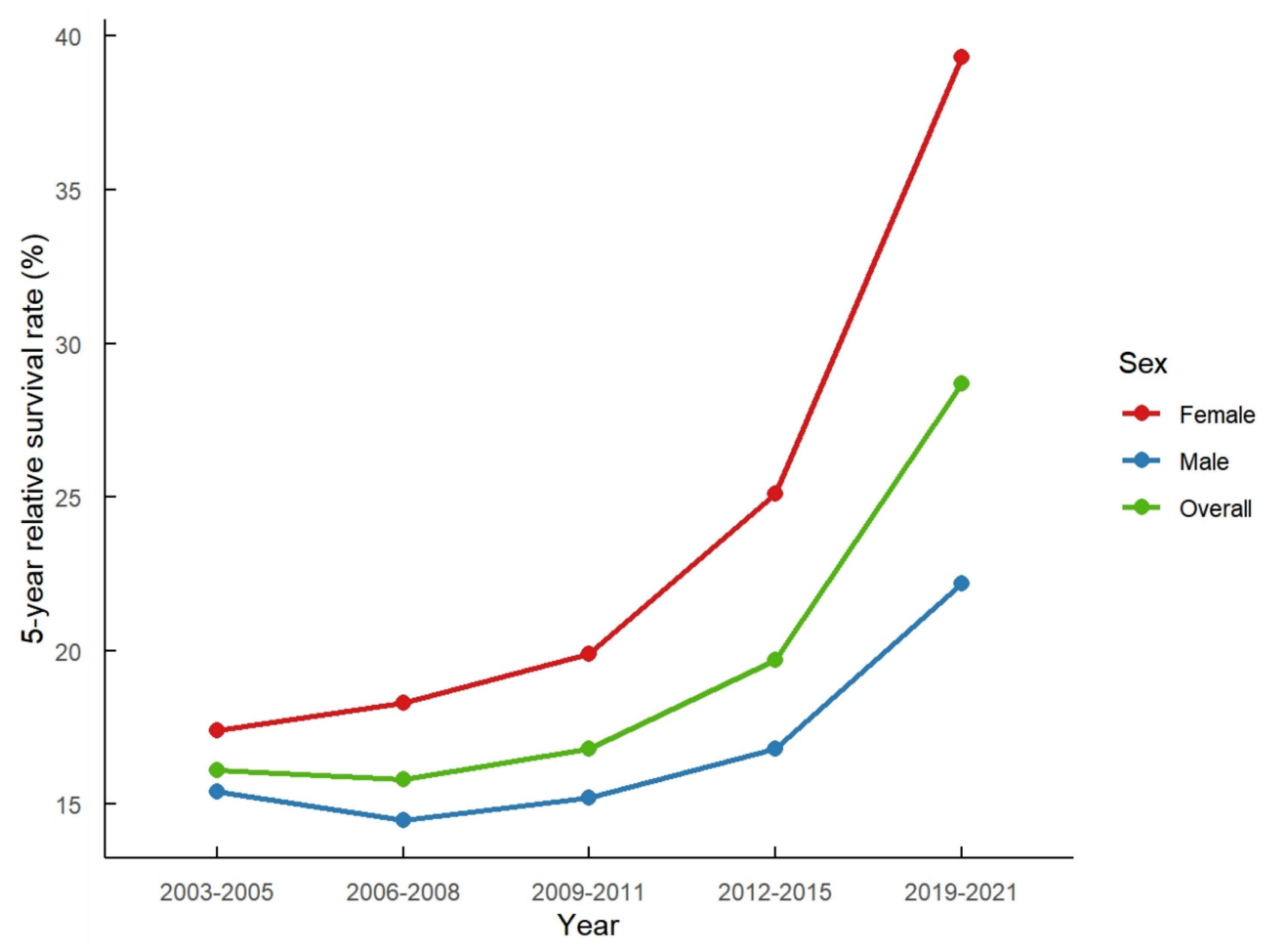
Trends in age-standardized 5-year relative survival rates for lung cancer in China from 2003 to 2021, overall and by sex. The data are derived from National Cancer Center of China (62).

LCINSF exhibit superior survival outcomes versus smokers. A Japanese cohort found that females with non-small cell lung cancer (NSCLC) had a 61.0% 5-year survival rate, 20.2% higher than males (61). Chinese registries showed that the female age-standardized survival rate was 39.3% vs. males’ 22.2%, with greater temporal improvement (55.7% vs. 30.6%) (Figure 2) (62). U.S. data confirmed this trend: NSF with NSCLC had a 64% 5-year survival rate vs. smokers’ 56% (*p* = 0.031), most pronounced in stage I (75% vs. 62%, *p* = 0.02) (63).

## 3 Risk factors

### 3.1 Genetic and family history of lung cancer

Genomic studies indicate that LCINSF has a distinct molecular profile. KRAS mutations are more frequent in adenocarcinomas from never-smokers in North America and Europe, whereas epidermal growth factor receptor (EGFR) and tumor protein p53 (TP53) mutations are more prevalent in East Asian never-smokers (64). Genome-wide association studies (GWAS) have further shown substantial interethnic variation in susceptibility loci, including differences in the number of identified loci and in the direction and magnitude of effect (Table 1) (65–74). For example, variants at 5p15.33 (CLPTM1L-TERT) appear protective in European populations but confer increased risk in East Asian populations (66, 67). By contrast, some loci, such as rs7962469 at 12q13.13 and rs12265047 at 10q25.2, have shown more consistent effects across ancestries (72). In Chinese never-smoking females, a 21-single nucleotide polymorphism (SNP) polygenic risk score (PRS) identified a high-risk group with a 2.09-fold higher incidence of lung cancer, corresponding to a PAF of 17.9% (Table 2) (74).

**Table 1:** Comparison of genome-wide association studies (GWAS) on lung cancer susceptibility in LCINSF in Europe, East Asia and Africa.

Country	Chromosomal region	Genetic loci	SNP (rsID)	OR 95%CI	References
Europe	13q31.3	GPC5	rs2352028	1.46 (1.26–1.70)	Li et al. (71)
	5p15.33	CLPTM1L-TERT	rs31490	0.77 (0.77–0.82)	Hung et al. (67)
			rs380286	0.77 (0.72–0.82)	
			rs4975616	0.78 (0.73–0.83)	
	10q25.2	VT1A	rs12265047	0.63 (not reported)	Li et al. (72)
	12q13.13	ACVR1B	rs7962469	1.12 (not reported)	
East Asia	5p15.33	CLPTM1L-TERT	rs2736100	1.54 (1.41–1.68)	Hsiung et al. (66)
	3q28	TP63	rs10937405	0.82 (0.76–0.88)	Hosgood et al. (65)
	10q25.2	VTI1A	rs7086803	1.28 (1.21–1.35)	Lan et al. (70)
	6q22.2	ROS1, DCBLD1	rs9387478	0.85 (0.81–0.90)	
	6p21.32	HLA class II region	rs2395185; rs28366298	1.17 (1.11–1.23)	
	2p16.3	NRXN1	rs10187911	1.37 (1.23–1.51)	Kim et al. (69)
	5q32	CSF1R	rs10079250	1.85 (1.18–2.89)	Kang et al. (68)
	3q28	TP63	rs7631358	1.82 (1.10–3.03)	
	19p13.3	CIR1	rs13009079	0.53 (0.34–0.83)	
	6p21.1	DQ141194	rs7741164	1.17 (1.12–1.22)	Wang et al. (73)
	9p21.3	/	rs72658409	0.77 (0.72–0.84)	
	12q13.13	ACVR1B	rs11610143	0.89 (0.85–0.92)	
	5q11.2	PDE4D	rs1498606	1.15 (1.09–1.20)	Wei et al. (74)
	10q25.2	VT1A	rs12265047	0.77 (not reported)	Li et al. (72)
	12q13.13	ACVR1B	rs7962469	1.18 (not reported)	
Africa	10q25.2	VT1A	rs12265047	0.63 (not reported)	Li et al. (72)
	12q13.13	ACVR1B	rs7962469	1.74 (not reported)	

LCINSF, lung cancer in never-smoking females.

**Table 2:** Summary of risk factors for lung cancer among never-smoking females.

Risk factor	Exposure measure	OR/RR/HR (95%CI)		Evidence Level (Asian|Western)	P_e_ (%)	PAF (%)
		Asian	Western			
Genetic variants	PRS top 20%	2.09 (1.56–2.80)	/	2b	20 (China)	17.9 (China)^a^
Family history of lung cancer	First-degree-relative lung cancer	1.50 (1.29 1.75)	2.58 (1.35–4.94) (U.S.)	2b|3b; 3a	7.8 (China)	3.8 (China)^a^
					3.25 (Korea)	
			1.38 (1.07–1.78) (Europe)			1.6 (Korea)^a^
						1.0 (Japan)^a^
					2.0 (Japan)	
Secondhand smoke	≥20 pack-year	1.40 (1.08–1.82)	1.61 (1.00–2.58) (U.S.)	2a|2b	/	18.0 (China)^b^
					/	
					30.5 (Japan)	
						20.5 (Korea)^b^
						10.9 (Japan)^a^
Air pollution (PM2.5)	10 μg/m^3^ increase	1.10 (1.04–1.17)	1.30 (1.01–1.68)	2b|2b	/	33.6 (China)^b^^,^ ^c^
					65.2 (Korea)	
						6.1 (Korea)^a^
					/	
						7.5 (Japan)^b^
Occupational exposure	Exposed to asbestos	1.70 (1.31–2.21)	/	3a; 2b|2a	13.3 (China)	8.5 (China)^a^
	Low-level radon-exposed (<118.44 working level month)		Not significant			
					/	/
			Not significant		/	/
		5.75 (1.26–26.12)				
		11.66 (3.24–42.05)				
	High-level radon-exposed (≥118.44 working level month)					
History of pulmonary disease	TB	2.23 (1.38–3.61)	1.90 (1.45–2.50)	2a|2a	15.0 (China)	20.0 (China)^a^
	Pneumonia		1.36 (1.10–1.69)			
					7.9 (Korea)	11.7 (Korea)^a^
					/	
			1.35 (1.12–1.63)			/
		1.84 (1.37–2.46)				
	COPD					
		2.67 (2.09–3.40)				
Cooking oil fumes	Cooking oil fumes exposure	1.74 (1.57–1.94)	/	3a	/	/
Household coal use	Indoor coal smoke exposure	2.93 (1.40–6.12)	2.15 (1.61–2.89)	3a | 3a	/	/
Hormonal & reproductive factors	Menarche at ≤11 years	Not significant	1.21 (1.09–1.34)	2b | 2b	/	/
			1.18 (1.06–1.31)			
			Not significant			
	Menopause at ≤46 years	Not significant				
	Oral contraceptive use	1.16 (1.02–1.33)				

PRS, polygenic risk score; TB, tuberculosis; COPD, chronic obstructive pulmonary disease; P_e_, prevalence of exposure; PAF, population attributable fraction; / indicates no evidence available. Evidence level follows the Oxford Centre for Evidence-Based Medicine (OCEBM) classification system.

a PAF was calculated by formula PAF = P(RR−1)/[P(RR−1) + 1], P_e_ was derived from the National Lung Cancer Screening Program’s non-smoking population, where females comprise 70%, Korea National Health and Nutrition Examination Survey (KNHANES), and Japan Public Health Center (JPHC).

b PAF was derived from published literature.

c The 33.6% PAF reflects the entire never-smoker population, not female never-smokers exclusively.

Overall, GWAS findings in NSF show limited across population overlap, with most loci exhibiting relatively modest effect sizes (OR <2). However, key limitations remain, including insufficient cross-ethnic validation, incomplete functional characterization of identified SNPs, and potential confounding from gene–environment interactions. Future research should prioritize multi-ancestry cohort analyses and mechanistic studies to elucidate causal pathways.

Family history is a practical marker of inherited susceptibility and shared environmental exposures. In a Chinese prospective cohort of 547,218 never-smoking females, a first-degree relative (FDR) history of lung cancer was associated with a 1.50-fold increased risk, with stronger associations observed as the number of affected relatives increased (11). Similar findings have been reported in Japanese and Korean populations (7, 10), and studies have generally shown stronger effects in females than in males (8, 10). Western studies also support this association: a U.S. study linked early-onset cancer in an FDR to a 2.58-fold higher risk in never-smoking females (9), and a European study reported an OR of 1.38, with stronger associations when onset occurred before age 50 years (6). Family history should therefore be viewed as a robust risk indicator, although it likely reflects both inherited predisposition and shared exposures rather than a purely genetic effect.

### 3.2 Environmental risk factors

#### 3.2.1 Shared risk factors

##### 3.2.1.1 Secondhand smoke

The IARC and the U.S. National Institutes of Health have classified secondhand smoke (SHS) as a Group 1 carcinogen. Several meta-analyses have reported the lung cancer risk of SHS exposure among NSF (12–14, 16). A meta-analysis by Ni et al. reported a pooled relative risk of 1.40 (95% CI: 1.08–1.82) for lung cancer associated with SHS exposure and demonstrated a dose–response relationship based on exposure metrics reported in the included studies, including exposure duration (<20 vs. ≥20 years), exposure intensity (<10 vs. ≥10 cigarettes/day), and cumulative exposure (<20 vs. ≥20 pack-years) (14). A meta-analysis by Kim et al. demonstrated higher lung cancer risk from SHS exposure in Asian (OR = 1.33, 1.10–1.61) than in Western (OR = 1.13, 1.01–1.25) (13). Interestingly, a large prospective cohort study from the U. S. involving 76,304 females revealed that while SHS overall did not significantly increase lung cancer risk among NSF, those with long-term household exposure (≥30 years) showed a borderline elevated risk (HR = 1.61, 95% CI: 1.00–2.58) (18). In that study, SHS duration was primarily quantified based on self-reported years of exposure in the household environment, which helps explain why the effect estimate was more evident for prolonged cumulative exposure than for broader binary exposure categories.

The PAF of SHS exposure varies across Asian countries. A study in China revealed that approximately 16% of lung cancer cases among never-smokers may be attributable to SHS. This is slightly higher among females (approximately 18%) (75). Japan showed a more moderate PAF of 10.9%, and Korea a substantially higher PAF of 20.5% (Table 2) (15, 54).

Overall, the association between SHS exposure and lung cancer risk in NSF is supported by multiple epidemiological studies, although the magnitude of association varies across populations. Interpretation should remain cautious because SHS exposure is often self-reported, and residual confounding by household environment, socioeconomic status, and co-exposures cannot be excluded.

##### 3.2.1.2 Air pollution (PM_2.5_)

Fine particulate matter (PM2.5), classified as a Group 1 carcinogen by IARC in 2013, penetrates deep into the lungs and bloodstream, triggering oxidative stress and chronic inflammation that drive carcinogenesis. Global analyses reveal an increasing PM2.5-attributable lung cancer burden, particularly among reproductive-aged females in middle SDI regions and East Asia, while high SDI regions show declining trends (76, 77). Epidemiological evidence demonstrates robust exposure-response relationships: the China Kadoorie Biobank study found a 10% increased lung cancer risk per 10 μg/m^3^ PM2.5 increment in never-smokers (HR = 1.10, 95% CI: 1.04–1.17) (20), while U.S. data showed a 30% higher mortality risk at equivalent exposure levels in NSF (HR = 1.30, 95% CI: 1.01–1.68) (19). Notably, achieving the WHO PM2.5 guideline (10 μg/m^3^) could prevent approximately one-third (33.6%) of lung cancer cases in non-smoking populations, underscoring the substantial preventive potential of air quality regulation (20).

Emerging evidence further suggests that PM2.5 may promote lung tumorigenesis by fostering a pro-inflammatory microenvironment that expands pre-existing clones harboring oncogenic driver mutations, such as EGFR. This “initiation-promotion” model provides crucial mechanistic insight into the role of air pollution in lung cancer pathogenesis among never-smokers and offers a plausible explanation for the particularly high prevalence of EGFR-mutant lung cancer observed in Asian females (78).

Compared with several other putative risk factors, ambient air pollution particularly long-term PM2.5 exposure, has relatively consistent epidemiological support and plausible biological mechanisms. However, heterogeneity in exposure assessment methods and regional pollution patterns may partly explain variation in reported effect sizes.

##### 3.2.1.3 Occupational exposure

Multiple occupational carcinogens including asbestos, radon, beryllium, chromium, cadmium, nickel, silica, soot, and coal dust are established lung cancer risk factors, with asbestos, arsenic compounds, and radon-222 classified as Group 1 carcinogens by IARC.

Existing studies on occupational lung cancer risks have rarely focused on NSF (5). Nevertheless, prior research indicates that occupational exposure to silica, asbestos, inorganic arsenic, chromium, nickel, tar, and radon elevates lung cancer risk across populations. A meta-analysis found a 70% increased risk (OR = 1.70, 95% CI: 1.31–2.21) in non-smoking workers exposed to asbestos compared to unexposed individuals (28). Among Chinese tin miners, cumulative radon exposure demonstrated a dose–response relationships: low-level exposure (<118.44 working level months) increased lung cancer risk 5.75-fold (95% CI: 1.26–26.12), while high-level exposure (≥118.44 working level months) raised risk 11.66-fold (95% CI: 3.24–42.05) (27). Notably, a meta-analysis of primarily Western non-smoking populations found no significant association between residential radon exposure and lung cancer risk in females, contrasting with elevated risk in males, suggesting potential sex-based differences in susceptibility (26).

The reported effect sizes for certain occupational exposures are larger than those observed for SHS or ambient air pollution; however, such exposures are typically less prevalent in the general female population. Therefore, their population-level contribution may be smaller even when the individual-level risk is high. This distinction between relative risk and public health burden is important when prioritizing prevention.

##### 3.2.1.4 History of pulmonary disease

A history of pulmonary diseases, including tuberculosis (TB), pneumonia, and chronic obstructive pulmonary disease (COPD), has been implicated in elevating lung cancer risk among NSF. Current evidence on pulmonary disease history and LCINSF remains limited and inconsistent.

Two large meta-analyses demonstrate that while pre-existing pulmonary diseases collectively elevate lung cancer risk, TB and pneumonia consistently emerge as significant independent risk factors for NSF (21, 22). A cohort study from South Korea found that the risk of lung cancer in never-smokers with COPD was 2.67-fold higher (95% CI: 2.09–3.40) compared to those without COPD (23, 25).

Smoking and air pollution exposure elevate lung cancer risk in individuals with a history of pulmonary diseases, with chronic inflammation and parenchymal lung injury implicated as underlying mechanisms (79). Studies in southern China suggested that COPD, chronic bronchitis, and emphysema correlate more strongly with lung cancer in smokers than in never-smokers (24). A CKB-based study revealed that COPD mediated 22.8% of PM2.5’s effect on lung cancer incidence among never-smokers. After adjusting for COPD, the risk estimate of PM2.5 for never-smokers showed a slight decline, suggesting that COPD serves as one important pathway in PM2.5—induced carcinogenesis (20). However, most risks remained driven by PM2.5’s direct effects (e.g., DNA damage, inflammatory microenvironment) (78), underscoring the necessity of air pollution control.

#### 3.2.2 Unique risk factors

##### 3.2.2.1 Indoor air pollution

High-temperature cooking generates carcinogenic cooking oil fumes (COFs), classified by IARC as Group 1 indoor carcinogens containing polycyclic aromatic hydrocarbons (PAHs) and aldehydes. COFs have been associated with increased lung cancer risk in never-smoking females, particularly in East Asian studies and in settings with poor kitchen ventilation.

Case–control studies demonstrate strong dose–response relationships between COFs exposure and lung cancer risk in Chinese NSF (30, 34). Chen et al. (30) reported a 3.17-fold increased risk (95% CI: 1.34–7.68) for the highest exposure group (>160 time-years), while Yu et al. (34) found a striking 34-fold risk (95% CI: 7.16–161.39) at >200 dish-years, with methodological differences potentially explaining the variance. This difference may stem from the sensitivity differences in exposure assessment methods and the higher exposure levels due to the lower usage rate of fume extractors in Hong Kong. The results should be interpreted with caution. Cooking methods show differential risks: deep-frying (OR = 2.56, 95% CI: 1.31–5.00) > pan-frying (OR = 1.47, 95% CI: 1.27–1.69) > stir-frying (OR = 1.12, 95% CI: 1.07–1.18). Importantly, fume extractors reduce risk by 52.6%, highlighting ventilation’s protective role (33).

Household coal use is a significant global risk factor for lung cancer, with elevated risks particularly evident in Asian populations and NSF. A meta-analysis encompassing populations from Asia, Europe, North America, and Africa demonstrated that household coal use significantly increased the risk of lung cancer (OR = 2.15, 95% CI: 1.61–2.89). Among NSF, those exposed to coal smoke exhibited a 2.93-fold higher risk of lung cancer compared to unexposed individuals (OR = 2.93, 95% CI: 1.40–6.12). China (including Taiwan) exhibited an even higher risk (OR = 2.27, 95% CI: 1.65–3.12), with significant regional variations: the highest risk was observed in southwestern/southern regions, followed by northern and northeastern areas (29, 31, 33). The installation of chimneys can reduce lung cancer incidence and mortality by 35–56% (32), emphasizing the importance of clean energy transitions and ventilation improvements.

Importantly, the relevance of cooking oil fumes and household coal combustion is not uniform across populations. These exposures are likely to be especially important in certain East Asian and low- and middle-income settings, but their contribution may be substantially smaller in regions with different cooking practices, cleaner fuels, and better household ventilation. Therefore, these risk factors should be interpreted primarily as context-dependent rather than universally dominant determinants of LCINSF.

##### 3.2.2.2 Hormonal and reproductive factors

Hormonal and reproductive factors may constitute potential risk determinants for LCINSF, though current evidence remains inconclusive. Reproductive factors and exogenous hormone exposure demonstrate inconsistent associations with lung cancer risk across Eastern and Western populations. Early findings from a meta-analysis study covering females from Europe, Asia, and North America showed no significant association between oral contraceptive use and LCINSF risk (OR = 1.10, 95% CI: 0.93–1.30) (36). However, recent large-scale studies demonstrate geographical variations. Preliminary data from the UK Biobank cohort indicated modest associations between early menarche (HR = 1.21, 95% CI: 1.09–1.34), early menopause (HR = 1.18, 95% CI: 1.06–1.31), and lung cancer risk, though residual confounding cannot be ruled out (37). In contrast, a Chinese cohort of NSF found no significant associations between lung cancer risk and reproductive factors such as age at menarche, parity, age at first birth, or age at menopause. However, oral contraceptive use was associated with a significantly increased risk (HR = 1.16, 95% CI: 1.02–1.33) compared to never-users (35).

Nevertheless, these findings are derived from observational studies with heterogeneous populations and limited hormonal measurements. Future research should prioritize prospective designs with standardized endocrine assessments to address these limitations.

### 3.3 Geographic heterogeneity and comparative interpretation of risk factors

The relative importance of LCINSF risk factors differs markedly by region. Family history and PM2.5 appear broadly relevant across populations, whereas SHS, occupational exposures, and prior pulmonary disease are likely important globally but vary in exposure prevalence and effect size. By contrast, COFs and household coal use are strongly context-dependent and probably contribute disproportionately in parts of Asia and other settings where these exposures remain common.

This heterogeneity has important implications. Etiologically, pooled estimates should not be interpreted as evidence that a given exposure has equal importance in all settings. From a public health perspective, prevention and screening strategies should be adapted to local exposure profiles rather than extrapolated directly from one region to another.

### 3.4 Risk prediction models for lung cancer among NSF

Risk prediction models help identify high-risk non-smoking populations for lung cancer screening by quantifying individual probabilities. Chinese studies have developed models incorporating demographic and environmental factors. Guo et al.’s (80) nomogram for NSF - integrating age, chronic respiratory disease history, first-degree relative lung cancer history, menopausal status, and benign breast disease history - achieved an AUC of 0.762 for 1-year prediction. Similarly, the NCC-LCm2021 model, incorporating age, sex, BMI, family history, and chronic respiratory diseases, yielded an AUC of 0.698 in validation (81). Ma et al.’s (82) model, expanded to nine covariates from the China Kadoorie Biobank, reached an AUC of 0.759 in never-smokers. These models demonstrate the value of comprehensive risk stratification for lung cancer screening in non-smoking populations.

In addition, Western lung cancer risk prediction models (e.g., PLCOm2012, LCRAT, LCDRAT) exhibit limited generalizability to Asian non-smoking populations. A pooled analysis of 186,458 Asian ever-smokers revealed moderate discrimination (AUC: 0.68–0.71) but systematic underestimation of risk in those with <10 pack-years or prolonged smoking cessation (83). The Shanghai models, tailored to Asian populations, improved calibration while maintaining comparable discrimination (AUC: 0.70–0.72), underscoring the necessity of population-specific models for optimizing LDCT screening in Asia (83). Key limitations include low female representation (10.3%) and lack of biomarker integration—critical areas for refinement through polygenic risk scores or radiomic features to enhance predictive performance.

Besides, along with the technological innovation, risk prediction models by integrating multi-dimensional data are worthy of anticipation. The Sybil model, a deep learning algorithm analyzing LDCT imaging features, achieves an AUC of 0.92 for 1-year lung cancer risk prediction and demonstrates robust performance in non-smoking populations (84). Similarly, integrating blood-based biomarkers significantly enhances predictive accuracy—a four-marker protein panel (4MP) combined with the PLCOm2012 model yields an AUC of 0.85 (95% CI: 0.82–0.88), outperforming current USPSTF screening criteria (85). Additionally, a 21-SNP polygenic risk score (PRS-21) modestly improves prediction accuracy in Chinese NSF (AUC increase from 0.697 to 0.711) (74). While these innovations demonstrate potential, further validation is needed in populations with low smoking exposure (<10 pack-years) and diverse ethnic backgrounds to ensure broader applicability.

### 3.5 Prevention and clinical implications

Prevention of LCINSF should extend beyond smoking cessation. Because many affected women never smoked, strategies must target modifiable environmental and occupational risks in specific settings. Population priorities include reducing secondhand smoke exposure, improving air quality, promoting cleaner household fuels, strengthening kitchen ventilation, and minimizing occupational exposure to carcinogens. Where indoor combustion and cooking exposures remain common, household and community interventions may yield meaningful benefits.

Prioritization should reflect local exposure patterns. For example, policies targeting ambient PM2.5 and smoke-free public environments are broadly relevant, while reducing cooking oil fume or coal exposure is important in Asian or low-resource settings. Context-specific approaches avoid overgeneralization and maximize impact.

From a clinical perspective, physicians should not equate “never-smoking” with “low-risk.” Clinical risk assessment in women who have never smoked should include a structured evaluation of secondhand smoke exposure, occupational history, household fuel use, cooking practices and ventilation, prior pulmonary diseases, and family history of lung cancer. Such an approach may improve recognition of individuals who warrant closer follow-up, further imaging evaluation, or inclusion in future risk-based screening frameworks. These findings also have implications for counseling. Clinicians can use the current evidence to advise patients on practical preventive measures, such as reducing long-term household SHS exposure, improving kitchen ventilation, using fume extractors during high-temperature cooking, transitioning to cleaner fuels when possible, and seeking medical evaluation for persistent respiratory symptoms even in the absence of smoking history.

Finally, from a research and health-system perspective, prevention and early detection should be linked through better risk stratification. Because broad LDCT screening of all never-smoking females is not currently evidence-based, a more realistic path forward may involve identifying subgroups with clustered risk factors, such as family history, chronic pulmonary disease, prolonged SHS exposure, or high regional pollution burden, who may derive greater net benefit from surveillance or future screening programs.

## 4 Screening

### 4.1 Controversy of LDCT screening for NSF

Two landmark RCTs—the National Lung Screening Trial (NLST) in the United States and the Nederlands-Leuvens Longkanker Screenings Onderzoek (NELSON) trial in Europe—established a 20–24% mortality reduction through LDCT screening (38, 39). However, these trials along with screening guidelines primarily targeted heavy smokers due to their inclusion criteria based on smoking history, thereby not adequately encompassing the never-smoking population. For instance, the USPSTF recommends annual LDCT for individuals aged 50–80 years with ≥20 pack-year smoking history, either current smokers or those having quit ≤15 years (86). Guidelines from China have pioneered a more inclusive risk stratification framework, incorporating additional exposure variables beyond smoking status (87).

Emerging Asian studies have revealed critical insights into the effectiveness of LDCT screening in never-smokers. A recent JAMA study reported comparable lung cancer detection rates between Chinese never-smokers (1.6%) and smokers (2.0–2.3%), with never-smokers showing superior early-stage detection (93.2% vs. 80.4%) (43). A meta-analysis of 13 Asian studies revealed equivalent diagnostic yield between NSF and high-risk former-smoking males (RR = 1.02, 95% CI: 0.94–1.11), alongside significantly higher early-stage detection (82.4% vs. 50.6%) and lower mortality (HR = 0.59, 95% CI: 0.42–0.83) in never-smokers (44). The Taiwan Lung Cancer Screening for Never-Smokers Trial (TALENT) in China implemented enhanced risk stratification incorporating NSF-specific factors, demonstrating a 2.6% baseline lung cancer detection rate within first-year follow-up, exceeding the 1.1% in the NLST (40). Higher detection rates of invasive lung cancer among NSF were also reported by the Female Asian Never-Smoker Screening Study (FANSS) and a Korean cohort study (41, 42). These findings suggest that LDCT can detect early-stage lung cancer in NSF and demonstrate the operational feasibility of screening in selected Asian cohorts (Table 3).

**Table 3:** Lung cancer screening study including never-smoking females.

Country	Author	Study design	Year	Sample	Proportion of never-smoking female	Inclusion criteria except for smoking	Outcome
China	Chang et al. (40)	Cohort study	2015–2019	12,011	73.83%	Family history of lung cancer; Passive smoking; History of TB or COPD; Cooking index ≥110; Cooking without using ventilation	Detection rate at baseline: 2.6%, 2.7% with a family history of lung cancer vs. 1.6% without a history
	Yang et al. (45)	Randomized controlled trial	2013–2014	6,717	Female: 52.75%	45–70 years of age and one of the following: smoke ≥20 pack-year; family history of cancer; Occupational exposure to carcinogenic agents; passive smoking; exposure to cooking oil fumes	Detection rate: 1.48% in LDCT screening arm and 0.32% in control arm
					Never-smokers: 23.46%		
	Zhang et al. (46)	Cohort study	2012–2018	15,686	Female: 71%	Employees who volunteered to take LDCT	Detection rate: 2.2% in non-smokers 1.4% in smokers
					Never-smokers: 91%		
South Korea	Kang et al. (42)	Cohort study	2003–2016	12,176	63.6%	Patients who underwent LDCT screening between May 2003 and June 2016	Detection rate: 0.45% in non-smokers and 0.86% in smokers
							Early-stage proportion: 92.7% in non-smokers and 63.6% in smokers
USA	Shum et al. (41)	Cohort study	2021–2023	201	100%	Female; 40–74; Asian; Never-smoker	Detection rate: 1.5%

TB, tuberculosis; COPD, chronic obstructive pulmonary disease; LDCT, low-dose computed tomography.

However, the higher detection rate of lung cancer may not necessarily translate into reduced mortality. These findings should be interpreted with caution. A higher detection rate and a greater proportion of stage I cancers do not, by themselves, demonstrate a true screening benefit. In never-smoking females, apparent advantages of LDCT screening may be influenced by several well-recognized epidemiological biases. Lead-time bias may create the illusion of longer survival simply by advancing the time of diagnosis without altering the natural course of disease. Length bias may preferentially detect slower-growing, less aggressive tumors that are more likely to be identified during periodic screening. In addition, overdiagnosis remains a major concern, particularly in populations with a relatively low baseline risk of lung cancer mortality, because LDCT may identify indolent lesions that would never have become clinically significant during a patient’s lifetime.

One prospective study reported by the National Cancer Center of China demonstrated a significant 31% reduction in lung cancer mortality among high-risk males (HR = 0.69, 95% CI: 0.52–0.91), though no significant association emerged in female participants (48). This gender-discrepant outcome finds reinforcement in Wang et al.’s study, which similarly failed to identify mortality benefits from one-off LDCT screening in non-smoking populations (49). Notably, Xu et al.’s investigation revealed substantial overdiagnosis of lung adenocarcinoma in Chinese populations attributable to LDCT screening, particularly among NSF. Overdiagnosis rates exhibited temporal escalation from 22% in 2011–2015 to 50% in 2016–2020 (50). A Korean cohort study found that never-smokers, when undergoing indiscriminate screening, face similar risks of overdiagnosis (regardless of gender) with limited benefit (47). Elevated detection rates alone, without demonstrated mortality benefit, may primarily reflect overdiagnosis and lead to unnecessary interventions, highlighting the need for rigorous evidence from randomized controlled trials with mortality outcomes before broader implementation can be justified. These findings fuel the ongoing international controversy regarding LDCT’s risk–benefit calculus in never-smokers.

Rather than direct evidence from RCTs in never-smokers, a systematic search of ClinicalTrials.gov using the terms ‘Lung Cancer’, ‘never-smoker OR nonsmoker OR nonsmoking’, and ‘Screening’ yielded no ongoing or completed RCTs. The current rationale for implementing LDCT screening in NSF predominantly derives from observational studies and extrapolations derived from randomized controlled trials (RCTs) conducted in smoking populations. Although existing data indicate analogous detection rates between never-smokers and smokers, several pivotal clinical questions remain unresolved. These include the intervention’s capacity to reduce mortality, overdiagnosis risk, and the determination of optimal screening frequencies. To address these evidence gaps, initial efforts should prioritize prospective cohort studies evaluating LDCT screening’s efficacy in reducing lung cancer mortality among never-smokers. Should the cohort data demonstrate significant efficacy, subsequent large-scale RCTs comparing LDCT screening versus no screening would be warranted. Such methodologically robust investigations are imperative to establish a definitive risk–benefit profile of LDCT screening in this distinct population.

In summary, LDCT screening in never-smoking females remains controversial. Existing studies suggest that screening can detect a substantial number of early-stage lesions, particularly in East Asian populations, but the absence of randomized evidence, the potential for lead-time and length biases, and the risk of overdiagnosis warrant a cautious interpretation. Future work should focus on risk-stratified screening strategies and on evaluating clinically meaningful endpoints, especially lung cancer mortality, quality of life, and screening-related harms.

### 4.2 Potential value of new screening techniques

Liquid biopsy, a minimally invasive approach analyzing circulating biomarkers, has emerged as a transformative tool for cancer detection and monitoring. Recent advancements in these techniques have demonstrated significant potential for the early detection of lung cancer, offering alternatives to traditional screening methods (88–98). Cell-free DNA (cfDNA) assays exhibit high sensitivity across cancer stages, with prospective studies reporting sensitivities ranging from 71% (Stage I) to 98% (Stage IV) and specificities up to 98% (88, 90, 93, 97). Similarly, miRNA-based panels, such as 6- and 14-miRNA signatures, achieve sensitivities of 74–80% and specificities exceeding 90% (89, 91), while protein biomarkers (e.g., 6-protein panel) show promise with 88.5% sensitivity and 82% specificity (Table 4) (94).

**Table 4:** Use of liquid biopsy for the early detection of lung cancer.

Techniques	Biomarker	Study type	Sensitivity by stage					Specificity	Trial identifier
			I	II	III	IV	All stages		
cfDNA		Prospective case–control	71	89	88	98	80	58.0	NCT04825834 (93)
		Case–control and prospective cohort	81	100	100	100	100	98	NCT06011694 (88)
		Prospective cohort	21.9	79.5	90.7	95.2	74.8	NA	NCT02889978 (90)
	609 genes	Prospective cohort	86.7				90.4	83.1	(97)
Circulating tumor cells (CTC)		Prospective cohort					26.3	96.2	NCT02500693 (92)
miRNA	6 micRNA	Case–control	74				80	80	(91)
	14-miRNA	Case–control and cohort	76.3				82.8	93.5	(89)
	24-miRNA	Prospective cohort					62.6–84.4	29.7–32.2	NCT03452514 (98)
	24-miRNA	Prospective cohort	89	75		89	87	81	NCT02837809 (96)
Proteins	6 proteins	Case control	66.2	78.6	90.0	94.3	88.5	82	(94)

Despite these promising results, several limitations must be addressed. Key challenges include the incomplete understanding of lung carcinogenesis and tumor biology, stringent requirements for standardized sample collection and processing, and the need for robust clinical validation in screening cohorts (88–94, 96–98). Additionally, demonstrating clinical utility, cost-effectiveness, and obtaining regulatory approval remain significant hurdles. Addressing these limitations will be crucial for translating liquid biopsy technologies into routine clinical practice and optimizing lung cancer screening protocols. Overcoming these challenges could significantly enhance screening efficacy, reduce false-positive and false-negative rates, and ultimately improve patient outcomes. The integration of these advanced techniques into clinical workflows holds great promise for revolutionizing early lung cancer detection (95).

## 5 Conclusion

In summary, lung cancer epidemic in NSF is worthy of vigilance, particularly in East-Asian population, and there is an urgent need to identify environmental and genetic risk factors for LCINSF based on high-quality cohort studies. Lung cancer screening for NSF is facing major challenges: a disconnect between screening guidelines and reality, omission of high-risk NSF as well as short-term benefits from early detection accompanied by potential overdiagnosis and harms. Current evidence gaps persist in LCINSF, including population-specific epidemiological data, high-quality cohort studies on risk factors, and targeted RCTs for screening. Exploratory studies should be encouraged, including the construction of risk prediction models based on risk factors for identifying LCINSF-specific high-risk groups, prospective evaluation of the effectiveness and drawbacks of LDCT screening, and the continued development of novel multi-omics and liquid biopsy technologies. Furthermore, establishing a comprehensive system for lung cancer prevention and control, including epidemiological surveillance, identification of risk factors, primary prevention, as well as screening and early detection, is essential for tackling the growing burden of LCINSF.
